# A Treatise on Dislocations and Fractures of the Joints

**Published:** 1851-07

**Authors:** 


ARTICLE III.

*A Treeatise on Dislocations and Fractures of the Joints:*—By Sir ASTLEY COOPER BART, F.R.S., Sergeant Surgeon to to the King, etc. A new edition much enlarged. Edited by BRANSBY B. COOPER, F.R.S., Surgeon to Guy's Hospital. With additional observations, and a Memoir of the Author. A new American edition: pp. 496: 8 mo. Philadelphia: Blanchard & Lea: 1851: (from the publishers through S. C. Griggs & Co., Chicago.)

No Surgical work has attained to a higher position of authority in Great Britain and America, than the treatise before us. The distinguished author, an interesting memoir of whose life is prefixed to this edition, has left behind him no better or more enduring monument to his ability and skill as a surgeon and teacher, than the work before us.

Its eminently practical character, its clear, plain, and forcible delineations of principles and practice are such, as to make it both interesting and useful. No practitioner of Surgery should be without a copy of it in his library.

